# Clinical improvement of functional mitral and tricuspid regurgitation following transcatheter aortic valve implantation with the NAVITOR VISION system: a case report

**DOI:** 10.1093/ehjcr/ytag479

**Published:** 2026-06-22

**Authors:** Akira Otani, Joshi Tsutsumi, Tatsuki Doijiri, Minako Hayakawa, Mimiko Tabata

**Affiliations:** Department of Cardiology, Yamato Seiwa Hospital, 9-8-2 Minamirinkan, Kanagawa 242-0006, Japan; Department of Cardiology, Yamato Seiwa Hospital, 9-8-2 Minamirinkan, Kanagawa 242-0006, Japan; Department of Cardiology, Yamato Seiwa Hospital, 9-8-2 Minamirinkan, Kanagawa 242-0006, Japan; Department of Cardiovascular Surgery, Yamato Seiwa Hospital, 9-8-2 Minamirinkan, Kanagawa 242-0006, Japan; Department of Cardiovascular Surgery, Yamato Seiwa Hospital, 9-8-2 Minamirinkan, Kanagawa 242-0006, Japan

**Keywords:** AS, TAVI, Residual MR, Residual TR, Navitor Vision, Case report

## Abstract

**Background:**

Concomitant mitral regurgitation (MR) and tricuspid regurgitation (TR) are frequently encountered in patients with severe aortic stenosis (AS) and are associated with adverse clinical outcomes after transcatheter aortic valve implantation (TAVI). However, the impact of transcatheter heart valve selection on post-procedural multi-valvular haemodynamics remains incompletely understood.

**Case summary:**

An 87-year-old woman with symptomatic low-flow, low-gradient AS complicated by moderate-to-severe MR and severe TR was admitted with refractory heart failure despite intensive medical therapy. Given her advanced age, frailty, and high surgical risk, transfemoral TAVI was performed using a 23-mm Navitor™ Vision valve (Abbott Structural Heart, Santa Clara, CA, USA).

Valve selection was guided by the presence of a small aortic annulus and the need to balance optimal haemodynamic performance with minimization of potential mitral valve interaction. The procedure was successfully completed without significant residual transvalvular gradient or paravalvular leakage. Following TAVI, relief of afterload was achieved, leading to improvement in functional MR and TR, with discontinuation of inotropic support. Follow-up echocardiography demonstrated a reduction in both MR and TR, accompanied by a decrease in right ventricular systolic pressure.

**Conclusion:**

We report a case of symptomatic low-flow, low-gradient AS with concomitant MR and TR that was successfully treated with TAVI using a Navitor Vision valve. In patients with small annuli and complex multi-valvular disease, careful transcatheter heart valve selection and procedural optimization may facilitate favourable haemodynamic outcomes and reverse remodelling.

Learning points

- Comprehensive Heart Team assessment and appropriate valve selection are essential in patients with a small annulus and multi-valvular disease who are at high surgical risk.
- Relief of afterload by TAVI can result in functional reduction of secondary mitral and tricuspid regurgitation.

## Introduction

Transcatheter aortic valve implantation (TAVI) is an established treatment option for elderly and high-risk patients with symptomatic severe aortic stenosis (AS).^1,2^ Moderate or greater mitral regurgitation (MR) and tricuspid regurgitation (TR) are frequently observed in this population and are associated with adverse prognosis when persistent after TAVI.^3–6^ However, the impact of transcatheter heart valve design on post-procedural MR and TR remains unclear.^6–8^ We report a case of symptomatic AS with MR and TR, both of which improved following TAVI in a patient with a small annulus and high surgical risk.

## Summary figure

**Figure:**
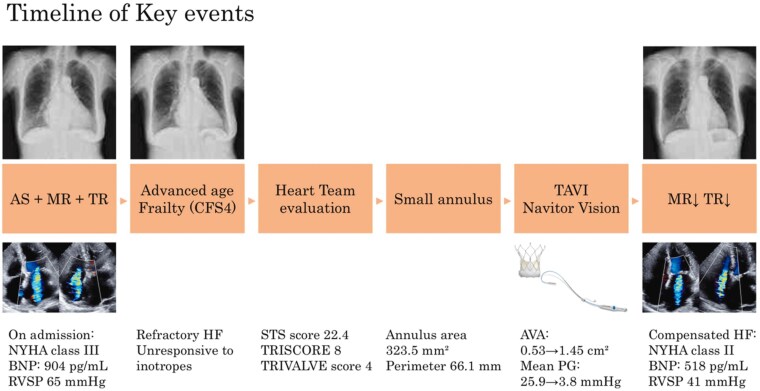


## Case presentation

An 87-year-old woman presented with progressive dyspnoea in June 2025. Her medical history included chronic heart failure, chronic kidney disease, paroxysmal atrial fibrillation, dyslipidaemia, and hypothyroidism. On admission, she was classified as New York Heart Association (NYHA) functional class III, with bilateral lower-extremity oedema. Laboratory tests revealed an elevated B-type natriuretic peptide level of 904 pg/mL (normal ≤18.4 pg/mL) and impaired renal function with a serum creatinine level of 1.7 mg/dL (normal range 0.45–0.82 mg/dL).

Electrocardiography revealed atrial fibrillation, and chest radiography revealed marked cardiomegaly (cardiothoracic ratio, 64.5%) with bilateral pleural effusion (*Figure 1*).

**Figure 1:**
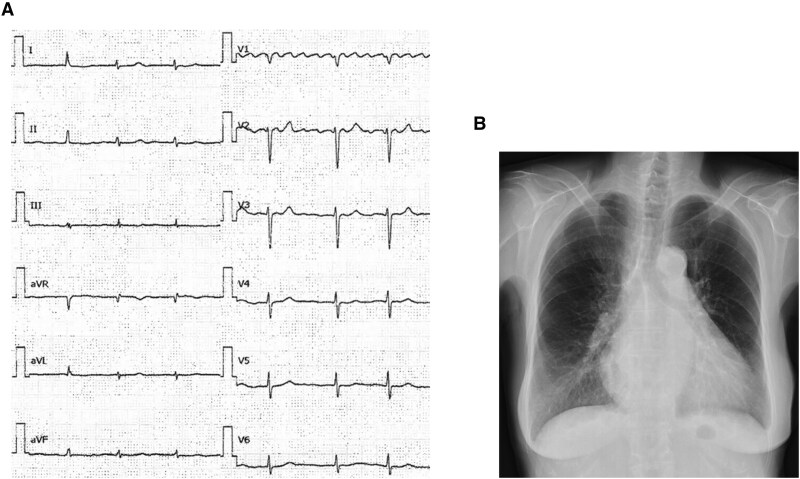
Baseline electrocardiography and chest radiography on admission. (*A*) Electrocardiography showed atrial fibrillation with a controlled ventricular rate of 80 beats per minute without significant ST–T segment abnormalities. (*B*) Chest radiography revealed cardiomegaly with a cardiothoracic ratio of 64.5% and mild pulmonary congestion.

Transthoracic echocardiography revealed an interventricular septal thickness of 10 mm and a posterior wall thickness of 9 mm, with a calculated left ventricular mass of approximately 108 g and a left ventricular mass index of 74 g/m^2^. The left ventricular end-diastolic and end-systolic diameters were 38 and 21 mm, respectively, with a preserved left ventricular ejection fraction of 74%, consistent with heart failure with preserved ejection fraction. Stroke volume and stroke volume index were 39.8 mL and 27.3 mL/m^2^, consistent with a low-flow state.

AS was assessed as moderate-to-severe based on echocardiographic parameters, with a peak transvalvular velocity of 3.3 m/s, a mean pressure gradient of 25.9 mmHg, and an aortic valve area of 0.53 cm^2^ (indexed aortic valve area, 0.36 cm^2^/m^2^). Moderate-to-severe functional MR and severe functional TR with annular dilatation were also noted, along with right atrial dilatation, mild right ventricular dilatation, pulmonary hypertension, and an estimated right ventricular systolic pressure of 65 mmHg (*Figure 2*; Supplementary material online, *Video S1*).

**Figure 2:**
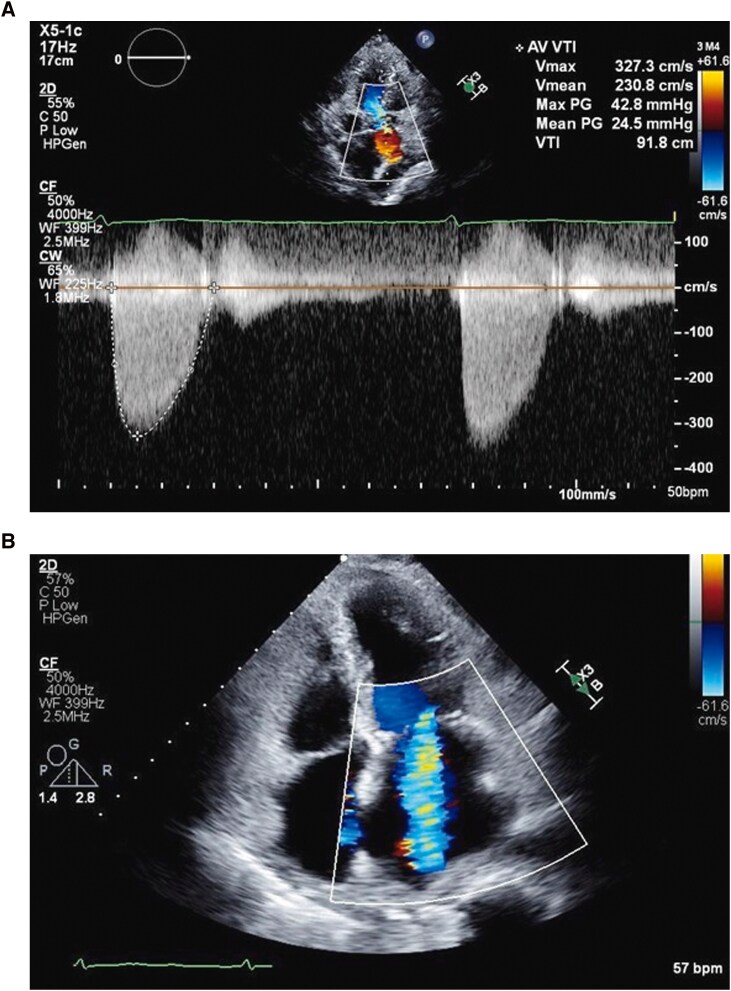

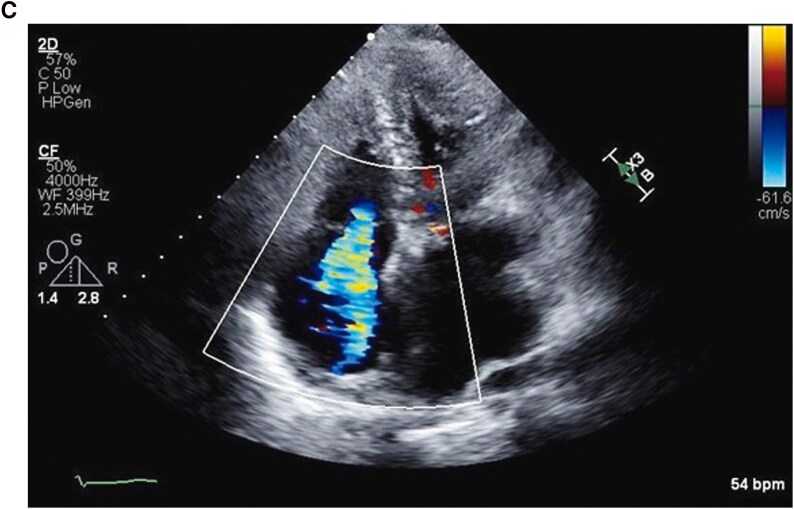
Transthoracic echocardiography before transcatheter aortic valve implantation. (*A*) Apical three-chamber continuous-wave Doppler imaging revealed a peak velocity of 3.3 m/s and a mean gradient of 25.9 mmHg. (*B*) Apical four-chamber view demonstrated moderate-to-severe functional mitral regurgitation. (*C*) Colour Doppler imaging showed severe tricuspid regurgitation.

The patient was hospitalized for heart failure in February 2025 when combined valvular disease was identified. Surgical intervention was deemed inappropriate because of her advanced age and frailty, and she was managed conservatively with diuretics to achieve temporary stabilization. However, during the current hospitalization, her heart failure remained refractory despite intensive medical therapy. On admission, she was taking dapagliflozin (10 mg), bisoprolol (2.5 mg), sacubitril/valsartan (200 mg), and esaxerenone (2.5 mg). Because of persistent congestion and signs of low cardiac output, oral azosemide (30 mg), tolvaptan (7.5 mg), intravenous furosemide (40 mg), and dobutamine (3 μg/kg/min) were administered during her hospitalization.

Given the limited response to pharmacological therapy, along with the patient’s advanced age and high surgical risk (Clinical Frailty Scale score 4, Society of Thoracic Surgeons score 22.4%, TRI-SCORE score 8, and TRIVALVE score, 4), the Heart Team determined that TAVI was the most appropriate treatment strategy for AS.

A comprehensive pre-procedural TAVI CT protocol was followed. The aortic annulus area was 323.5 mm^2^, with a perimeter of 66.1 mm. The mean sinus of Valsalva diameter was 28.9 mm (left coronary cusp 28.9 mm, right coronary cusp 28.9 mm, and non-coronary cusp 28.8 mm) (*Figure 3A* and *B*), and the sinotubular junction measured 23.2 × 25.7 mm. The aortic root angle was 46°, and the membranous septum length was 5.2 mm. The aortic valve Agatston score was 466. The right coronary height was 11.3 mm, and the left coronary height was 10.9 mm. Although mild coronary artery calcification was observed, no significant obstructive coronary artery disease was identified (*Figure 3C–F*). Despite the presence of chronic kidney disease, a reduced-contrast protocol was not followed because a comprehensive anatomical assessment was considered necessary for procedural planning. The total volume of contrast medium used for CT acquisition was 90 mL. Echocardiographic and CT findings alone do not fully meet the conventional criteria for severe AS. However, following a comprehensive Heart Team evaluation, an integrated assessment incorporating the presence of drug-refractory heart failure supported the decision to proceed with TAVI.

**Figure 3:**
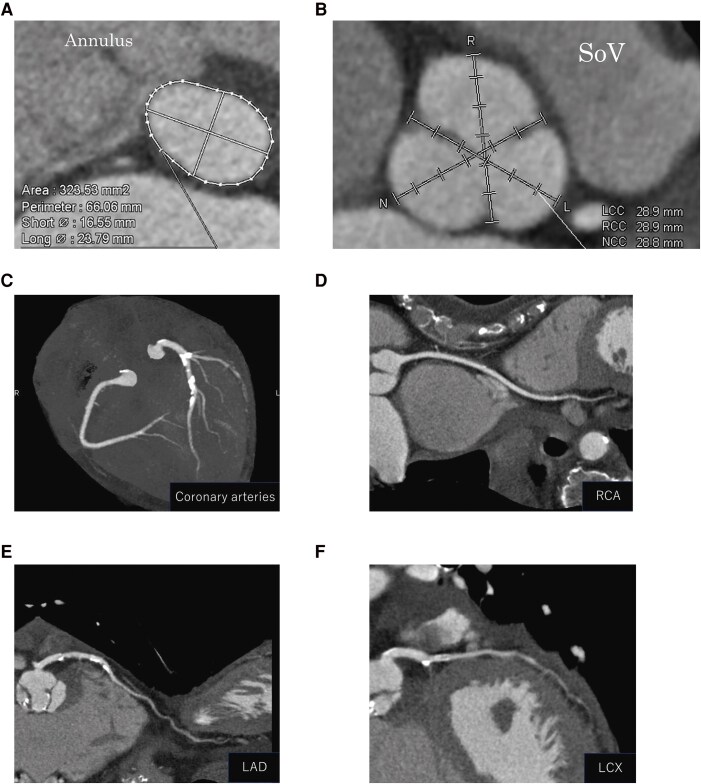
Pre-procedural contrast-enhanced computed tomography. (*A*) Measurement of the aortic annulus area and perimeter. (*B*) Axial view of the aortic root demonstrated the sinus of Valsalva. (*C*) Three-dimensional angiographic view of the coronary arteries. (*D–F*) Curved multi-planar reconstruction images of the right coronary artery, left anterior descending artery, and left circumflex artery.

Transfemoral TAVI was successfully performed via the right femoral artery using a Navitor^TM^ Vision valve (Abbott Structural Heart) (*Figure 4*). Post-procedural echocardiography demonstrated satisfactory prosthetic valve function, with a peak velocity of 1.3 m/s, a mean transvalvular pressure gradient of 3.8 mmHg, and an effective orifice area of 1.45 cm^2^, without evidence of paravalvular leakage. The patient was successfully weaned off the inotropic support, and intensified diuretic therapy was discontinued. She was discharged in stable condition (NYHA Class II). At 1 month, chest radiography showed resolution of congestion, and transthoracic echocardiography demonstrated moderate improvement in MR and TR (*Figure 5*; Supplementary material online, *Video S2*), with the right ventricular systolic pressure reduced to 41 mmHg, suggesting favourable post-TAVI haemodynamic remodelling.

**Figure 4:**
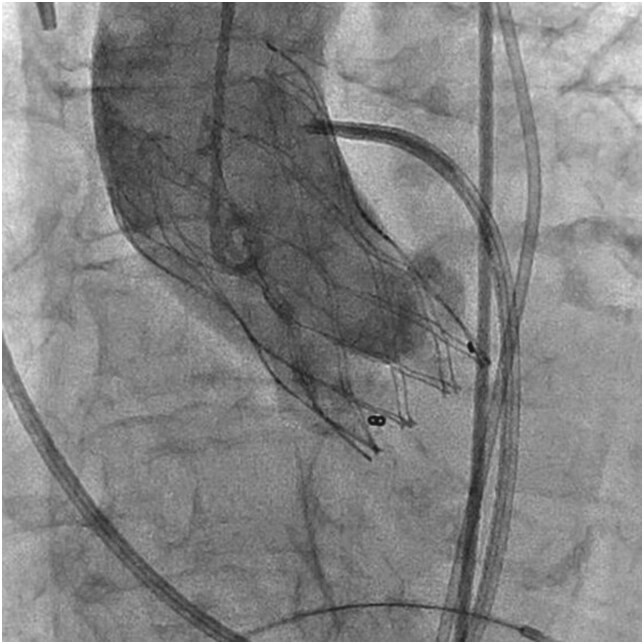
Transfemoral transcatheter aortic valve implantation using the Navitor Vision system. Fluoroscopic image showed the positioning of the 23-mm Navitor Vision valve.

**Figure 5:**
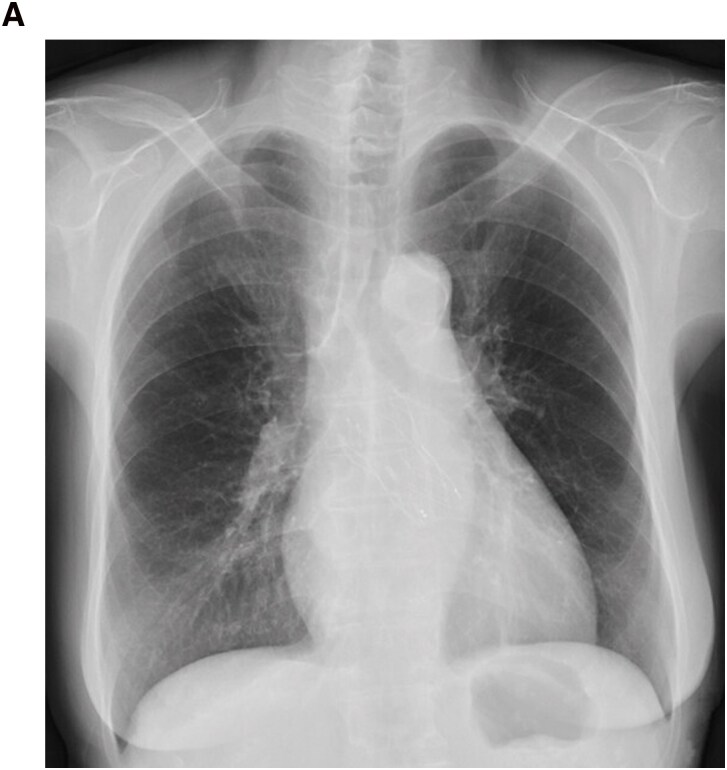

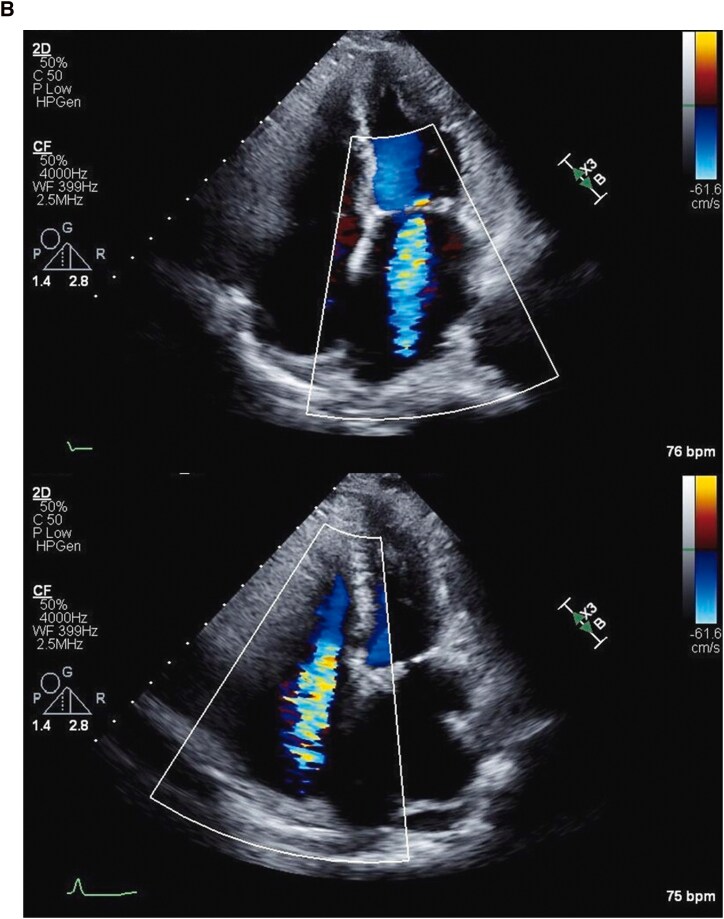
Post-procedural chest radiography and transthoracic echocardiography after TAVI. (*A*) Post-procedural chest radiography demonstrated a cardiothoracic ratio of 53.3%. (*B*) An apical four-chamber view demonstrated moderate mitral regurgitation and moderate tricuspid regurgitation.

## Discussion

Concomitant MR and TR are common in patients with severe AS and are associated with adverse outcomes when they persist after TAVI.^4–6^

The selection of the transcatheter heart valve is important for achieving favourable haemodynamics, particularly in patients with a small annulus. Balloon-expandable valves may result in higher residual gradients in small annuli, whereas self-expanding supra-annular valves generally provide larger effective orifice areas but are associated with higher rates of conduction disturbances and permanent pacemaker implantation.

In this case, valve selection required balancing favourable haemodynamics with the risk of mitral valve interference. A 20-mm Sapien valve was considered potentially suboptimal because of concerns about residual gradient in the small annulus, whereas the CoreValve/Evolut platform carried a potential risk of mitral valve interaction due to its flared frame extending towards the left ventricular outflow tract. The Navitor Vision valve was therefore selected because its relatively cylindrical frame configuration was considered less likely to interfere with the mitral valve while maintaining acceptable haemodynamic performance. The 23-mm valve size was selected according to CT-derived annular measurements and manufacturer recommendations (see Supplementary material online, *Figure S1*). Additionally, its radiopaque tantalum markers may facilitate accurate positioning and controlled high implantation, which may minimize mechanical interference with the mitral valve. These features may facilitate precise deployment in anatomically challenging cases with a complex valvular anatomy.

In the present case, device-specific characteristics may have contributed to favourable haemodynamics and reverse remodelling, resulting in the improvement of both MR and TR. The distinctive aspect of this case was the valve selection strategy aimed at balancing optimal haemodynamic performance with minimization of mitral valve interaction in a patient with a small annulus and complex multi-valvular disease.

As a single case report, these findings cannot be generalized, and long-term follow-up is needed. Nevertheless, this case highlights the importance of careful transcatheter heart valve selection and haemodynamic optimization during TAVI in patients with complex multi-valvular disease.

## Conclusion

We report a case of symptomatic low-flow, low-gradient AS with concomitant MR and TR that was successfully treated with TAVI using a Navitor Vision valve. In patients with small annuli and complex multi-valvular disease, careful transcatheter heart valve selection and procedural optimization may facilitate favourable haemodynamic outcomes and reverse remodelling.

## Supplementary Material

ytag479_Supplementary_Data

## Data Availability

The data underlying this article are available within the article.

## Lead author biography

Dr. Akira Otani is a cardiologist at Yamato Seiwa Hospital, Japan. He graduated from Tokyo Medical University and completed his residency and cardiology training at The University of Tokyo Hospital. He subsequently graduated from the Graduate School of The University of Tokyo. He is committed to improving patient outcomes through evidence-based practice and innovative therapies.

## Notes

**Consent:** Written informed consent was obtained from the patient for publication of this case and all accompanying images.
