# Supplementary material for: Clinical improvement of functional mitral and tricuspid regurgitation following transcatheter aortic valve implantation with the NAVITOR VISION system: a case report
Source: Eur Heart J Case Rep. 2026 Jun 22;10(7):ytag479. doi: 10.1093/ehjcr/ytag479 (PMC13344857; doi:10.1093/ehjcr/ytag479)
Supplement: ytag479_Supplementary_Data [file ytag479_supplementary_data.zip › Supplementary captions.docx]

**Supplementary Figure Legends**

**Supplementary Figure 1**

Manufacturer-recommended sizing chart for the Navitor Vision transcatheter aortic valve system based on computed tomography–derived aortic annular area and perimeter measurements. The chart illustrates the relationship between the annular dimensions and the recommended prosthesis sizes.

**Supplementary Video Legends**

**Supplementary Video 1**

(A)

Pre-procedural transthoracic echocardiography demonstrating moderate-to-severe mitral regurgitation in the apical four-chamber view.

(B)

Pre-procedural transthoracic echocardiography demonstrating severe tricuspid regurgitation in the apical four-chamber view.

**Supplementary Video 2**

(A)

Post-procedural transthoracic echocardiography showing moderate mitral regurgitation in the apical four-chamber view.

(B)

Post-procedural transthoracic echocardiography showing moderate tricuspid regurgitation in the apical four-chamber view.
