# Supplementary figures and images for: Clinical improvement of functional mitral and tricuspid regurgitation following transcatheter aortic valve implantation with the NAVITOR VISION system: a case report
Source: Eur Heart J Case Rep. 2026 Jun 22;10(7):ytag479. doi: 10.1093/ehjcr/ytag479 (PMC13344857; doi:10.1093/ehjcr/ytag479)

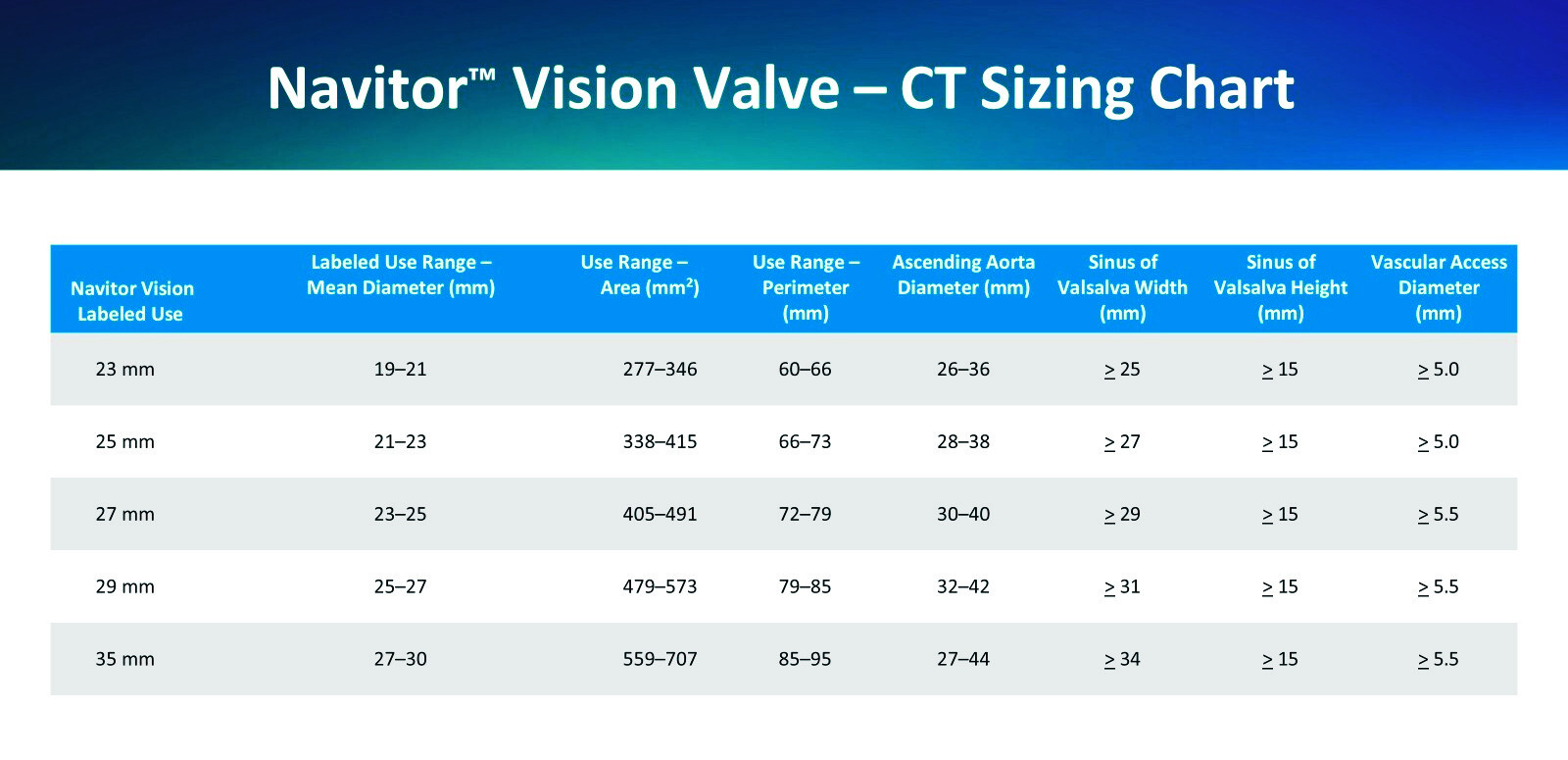

Supplement: ytag479_Supplementary_Data [file ytag479_supplementary_data.zip › Supplementary Figure 1.jpg]
